# Determining Asymmetry Thresholds in Anophthalmia/Microphthalmia Using a Three-dimensional Animated Model

**DOI:** 10.1097/IOP.0000000000002711

**Published:** 2024-06-27

**Authors:** Emiel J. Romein, Annabel L. W. Groot, Jelmer S. Remmers, Birgit Lissenberg, Annette C. Moll, Peerooz Saeed, Dyonne T. Hartong

**Affiliations:** * Department of Ophthalmology, Amsterdam UMC, University of Amsterdam, Amsterdam Orbital Center, Amsterdam, Netherlands; † Department Epidemiology and Data Sciences, Amsterdam UMC, Vrije Universiteit Amsterdam, de Amsterdam, Netherlands; ‡ Amsterdam UMC, Vrije Universiteit Amsterdam, Cancer Center Amsterdam, Amsterdam, The Netherlands

## Abstract

**Purpose:**

Congenital microphthalmia and anophthalmia are rare development disorders with underdevelopment of the orbital region, resulting in asymmetry of the face. No clear guidelines exist to determine when these deviations are acceptable.

**Methods:**

The face of a healthy 6-year-old child was three-dimensionally scanned. On this scan, we modeled various incremental degrees of facial asymmetries using three-dimensional modeling software. We modeled for smaller palpebral fissures, sunken eyes, and downward displacement of the eye. We also tested whether adjusting the vertical palpebral fissure height in relation to the horizontal palpebral fissure width affected perception. A total of 22 videos were created in which the model turned the head horizontally and vertically. We created a questionnaire asking raters how acceptable the face is, on a linear scale from 0 to 10.

**Results:**

Results showed a correlation between the degree of asymmetry and the acceptability score of the raters. Enophthalmos of ≥6 mm, palpebral fissure width ≤79% compared with the other eye, and 2 mm or more downward displacement of the eye resulted in a significant different acceptability score. The desire for correction was strongly increased when these thresholds were exceeded. Adjusting the vertical palpebral fissure height to the horizontal palpebral fissure width resulted in a worse acceptability score.

**Conclusion:**

A unilateral sunken eye (enophthalmos) of 6 mm or more, asymmetric horizontal palpebral fissure length of ≤79%, and a lower position of one eye of more than 2 mm resulted in unacceptable judgment. These data can be used to evaluate treatment outcome in children treated for congenital microphthalmia and anophthalmia.

## Abstract 2

Facial asymmetry thresholds for microphthalmia were determined. Horizontal palpebral fissure width ≤79%, enophthalmos >6 mm, and downward eye displacement >2 mm were deemed significantly unnatural. Results guide treatment decisions and outcome measurements for microphthalmia.

Facial symmetry has long been associated with beauty and facial attractiveness.

The face is central to interpersonal interaction. When identifying a face, the central triangle (eyes, nose, and mouth) appears to be more important than the outer contours.^1^ For facial recognition, the eyes contribute more than any other facial feature.^2,3^ Using eye-tracking technologies, scan paths have shown that the viewing patterns are focused on the facial deformity.^4–6^

Congenital microphthalmia and anophthalmia (MICA) are disorders where the ocular growth is affected. These conditions are rare developmental deficits of the eyeball in the embryonic^7^ or fetal stages, and cause a wide range of phenotypes from subtle developmental disorders to conditions where the eye is completely absent. The absence of a normal-sized eye is associated with growth retardation of the orbital structures and periocular soft tissues, causing small eyelids (microblepharon) and periocular volume deficiency. These asymmetries, even with the use of an ocular prosthesis, may be very noticeable, which can have a significant impact on the patient’s quality of life.^8^ Disproportionate growth between the skull and the affected orbit results in various facial asymmetries.^9^

Current treatment strategies aim to mimic the growth of a normally growing eye to prevent asymmetry when this process is disrupted. The fastest growth of the eye and orbit is in the early childhood, and they are almost fully grown at 5 years of age.^10^ Therefore, it is important to start treatment at a very early age to maximize growth stimulation of the affected side.^11–15^

Conformers are commonly used to stimulate eyelid growth by increasing the size periodically. This can also be done using inflatable silicone implants, self-expanding spheres/hemispheres, or pallets.^15–22^ Sometimes additional orbital surgery is performed to replace orbital volume deficit.

However, all faces remain with some degree of asymmetry after treatment. A distinction must therefore be made between a normal asymmetry and a disturbing asymmetry. To assess whether the degree of asymmetry is acceptable, we need to determine which level of deformation is acceptable and which is not.

There have been no studies that evaluated the characteristics of facial asymmetry in MICA specifically. Previous studies have focused on eyelid position (ptosis), blinking, and brow elevation in adult nonmicrophthalmic patients.^6,23,24^ It was found that the upper eyelid position was most sensitive, with an asymmetry threshold of 1 to 2 mm. A threshold of 3 to 4 mm was found for eyebrow elevation. A blinking delay of 33 msec was also distinguished as a notable asymmetry. These studies have been done using 2-dimensional (photographic) anthropometric methods. Better applicable anthropometric methods, however, make use of 3-dimensional technologies.^25,26^

No clear guidelines exist to assess whether correction for asymmetry in MICA is desirable. Our aim is to provide clear clinical guidelines to assess the severity of the asymmetry and when correction is beneficial for the perception of asymmetry, with the use of 3-dimensional (3D) animated models.

## METHODS

The face of a healthy 6-year-old child was scanned three-dimensionally with a handheld 3D scanner (Artec Leo 1.6, Luxembourg). We obtained written consent from the child’s guardian to use the facial scan for this research. The scanner has a high accuracy and measures to within 0.1 mm, but the 3D scanner has difficulty scanning thin and reflecting objects and therefore does not register the eyelashes, eyebrow hairs, and corneas. The generated point cloud was transcribed into a mesh. Next, we used meshmixer (version 3.5.474; Autodesk, San Rafael, CA, U.S.A.) to create a perfectly symmetrical face by mirroring one side of the face. Artificial corneas were then created in the 3D modeling software (Blender, Amsterdam, the Netherlands). This software uses raytracing to create realistic shadows and surface reflections. All this resulted in a perfectly symmetrical face with corneal reflexes, shadows, and surface reflections. The child and the parents gave consent to use the images for the purpose of this study. This study was exempted from ethical approval by the Ethics Committee and was performed according to the tenets of the Declaration of Helsinki.

**FIG. 1.**
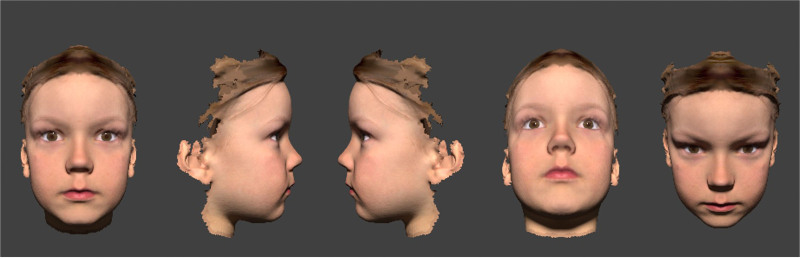
Standard model.

While creating the 3D models, we noticed that shadows and reflexes strongly affect the appearance. In particular, the incident light angle creates different aspects of what the affected eye looks like.^27^ We addressed this problem by creating animation videos where the head moves and therefore shifts shadows and corneal reflexes. The standard model without deviations is shown in Figure 1.

**Video 1.** 

On this standard model, various incremental degrees of facial asymmetries were modeled using the 3D modeling software. Based on our own perceptions of cosmetic appearance in our cohort of patients with MICA we modeled smaller palpebral fissures, enophthalmos, and caudal displacement of the eye, the latter with or without brow displacement. We chose to model only the OD because it is the side least perceptible to change^28^ and to be able to compare the left and right sides of the face.

**FIG. 2.**
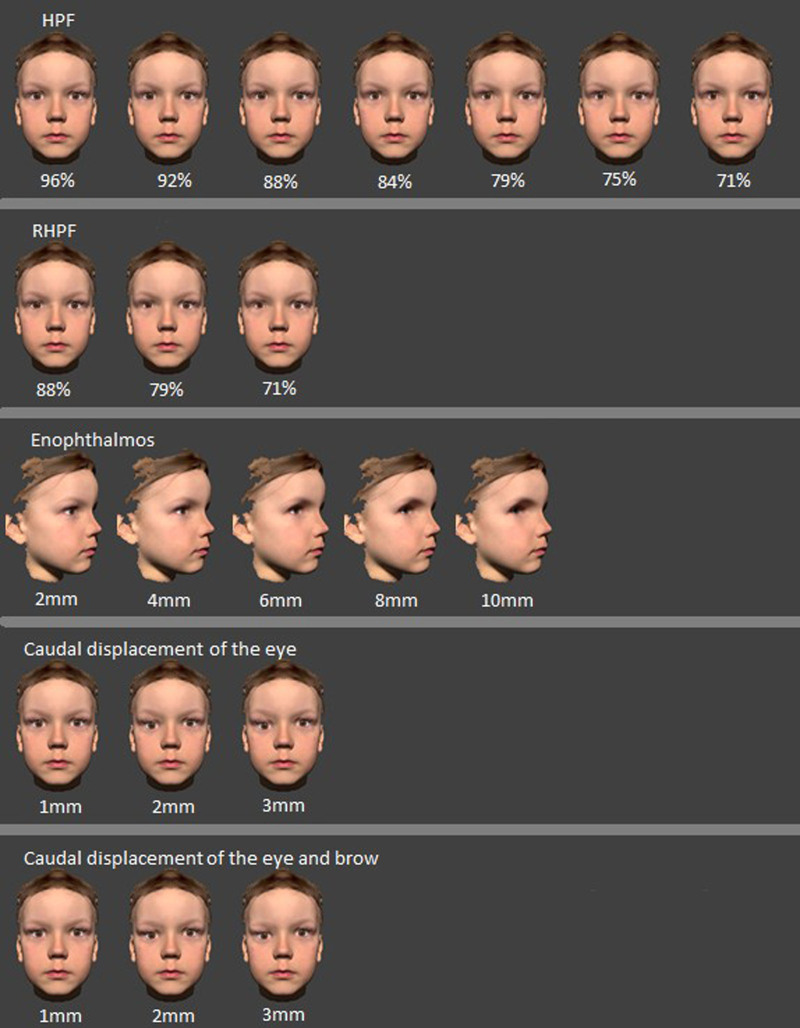
Deviations of the eye. HPF, horizontal palpebral fissure; RHPF, ratioed horizontal palpebral fissure, where the decrease in horizontal width equals a proportional decrease in the vertical length.

The resulting models are shown in Figure 2. The horizontal palpebral fissure (HPF) width in our standard model was 24 mm, and in the deviating models, we reduced this distance in 1 mm increments. This resulted in 7 scans with deviations from 100% to 71% symmetry values. As the palpebral fissure narrows, the shape of the eye changes to become rounder. To see if the change of shape affects the perception of asymmetry, 3 models were made while maintaining the horizontal to vertical palpebral fissure ratio of the standard model and named these the ratioed horizontal palpebral fissure (RHPF). The enophthalmos was created in decreasing steps of 2 mm, resulting in 5 models. The caudal displacement of the eyes was done in 3 steps with decreasing steps of 1mm. We made 2 models for each step, one with only caudal displacement of the eye and one with caudal displacement of the eye and the brow simultaneously.

A total of 22 animations were thus created with realistic shadows and reflections. (Fig. 2)

### Questionnaire

We created a questionnaire and asked how natural the face looks for each animation using a visual analog scale, which we transferred into a rating from 1 (not natural at all) to 10 (very natural). We additionally asked for each deviation whether the respondent would like to receive corrective treatment.

### Analysis

A linear regression analysis was done in SPSS version 17 for each type of deformation. When proven to be significant, subgroups were created for each level of deformation compared with all the other levels of deformation. When a significant difference was found for each level of deformation the best cutoff point was determined by the Akaike information criterion.

The paired *t* test was done to determine whether the ratio of the horizontal to the vertical fissure differed from the modified ratios (HPF vs. RHPF), and to determine if lowering of the brow together with the eye, affected judgment compared to lowering the eye alone.

## RESULTS

A total of 72 respondents answered the questionnaire, of which 20 were doctors, 24 were medical employees, and 28 were laypeople. Medical employees and doctors were mainly from our Department of Ophthalmology.

### Score of Acceptability

Caudal displacement was significantly different between each level of displacement (*p* < 0.001). Akaike information criterion was the lowest for a displacement of 2 mm, meaning this best fits the threshold (Table 1).

**TABLE 1.** Scores on the visual analog scale regarding facial asymmetry in caudal displacement of the eye, enophthalmos, and horizontal palpebral fissure width

	Mean score	*p* value compared to other levels*	AIC
Caudal displacement			
0 mm (standard)	9,3889	<0.001	
1 mm lower	8,6111	<0.001	1415
2 mm lower	3,8889	<0.001	**1187**
3 mm lower	3,3333	<0.001	1423
Enophthalmos			
0 mm (standard)	9,3889	<0.001	
2 mm deep	9,0000	<0.001	2222
4 mm deep	8,6111	<0.001	2097
6 mm deep	4,4444	<0.001	**1790**
8 mm deep	3,4444	<0.001	2030
10 mm deep	2,3889	<0.001	2180
Horizontal palpebral fissure width			
100% (24 mm) (standard)	9,3889	<0.001	
96% (23mm)	9,1111	<0.001	2733
92% (22mm)	8.0000	<0.001	2624
88% (21mm)	7.1667	<0.001	2568
84% (20mm)	7.4444	<0.001	2555
79% (19mm)	5.8333	<0.001	**2450**
75% (18mm)	4.5556	<0.001	2498
71% (17mm)	3.3333	<0.001	2641

Bold values highlight the lowest akaike information criteria. i.e. The value that best fits the model.

* The mean difference is significant at the 0.05 level.

AIC, Akaike information criterion.

Scores were given from visual analogue scale from 1 (not natural at all) to 10 (very natural).

Enophthalmos was significantly different between each level of displacement (*p* < 0.001). Akaike information criterion was the lowest for a displacement of 6 mm, meaning this best fits the threshold (Table 1).

Reduced horizontal palpebral fissure width was significantly different between each level of displacement (*p* < 0.001). Akaike information criterion was the lowest for a displacement of 79%, meaning this best fits the threshold (Table 1).

The paired *t* test differed inconsistently between the caudal positioning of the eye alone and the caudal position of the eye and brow. The mean score intersected at 1 mm and 3 mm the eye and brow resulted in a worse score compared to the eye alone. At 2 mm this resulted in a better score (Table 2).

**TABLE 2.** Scores on the visual analog scale for caudal displacement of the eye alone versus caudal displacement of eye and brow

Caudal displacement	Mean score of the eye alone	Mean score of the eye and brow	*p* *
1 mm lower	8.61	7.94	<0.026
2 mm lower	3.88	6.00	<0.000
3 mm lower	3.33	2.34	<0.001

* The mean difference is significant at the 0.05 level.

Scores were given from visual analogue scale from 1 (not natural at all) to 10 (very natural).

Significant lower scores were given for the RHPF compared to the horizontal palpebral fissure width (Table 3).

**TABLE 3.** Scores on the visual analog scale for the horizontal palpebral fissure vs the ratioed horizontal palpebral fissure

Displacement	Mean score of HPF	Mean score of RHPF	*p* *
88%	7.17	5.72	<0.000
79%	5.83	3.89	<0.000
71%	3.33	2.06	<0.001

* The mean difference is significant at the 0.05 level.

HPF, horizontal palpebral fissure; RHPF, ratioed horizontal palpebral fissure

RHPF width, where the decrease in horizontal width equals a proportional decrease in the vertical length.

Scores were given from visual analogue scale from 1 (not natural at all) to 10 (very natural).

### Desire for Correction

**FIG. 3.**
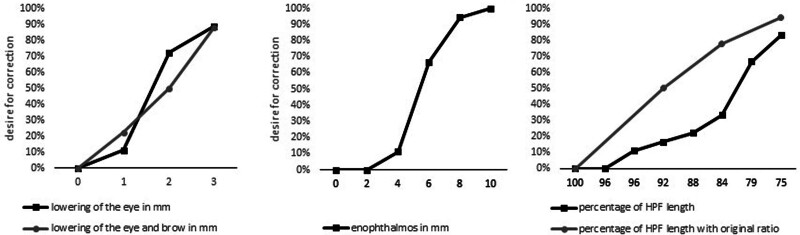
The desire for correction for each level of displacement. HPF, horizontal palpebral fissure; RHPF, ratioed horizontal palpebral fissure width, where the decrease in horizontal length is equals a proportional decrease in the vertical length.

The desire for correction scores (Fig. 3) showed an increasing desire for correction with increasing asymmetry. A large difference was found between the 1 and 2 mm caudal displacement of the eye. Here, the wish for correction went from 11% to 77%.

The desire for correction of enophthalmos took a big leap between 4 and 6 mm. Here it went from 11% to 67% (Fig. 3).

The desire for correction with a smaller horizontal palpebral fissure width increased gradually, but between 79% and 75%, it took a big step, from 33% to 66% (Fig. 3).

## DISCUSSION

In this study, we investigated when facial asymmetries in a child were found to be significantly unnatural with respect to horizontal eyelid length (≤79%), enophthalmos (>6 mm), and downward displacement of the eye (and brow) (2 mm). Results can guide in decision-making and defining outcome measurements in the treatment of congenital anophthalmia and microphthalmia.

This study measured asymmetry thresholds specific for microphthalmia. A similar threshold for HPF width was found in a previous study of our group.^29^ In this study, the HPF width cutoff point from mild to moderate disease was also around 80%, as it was judged by parents and physicians that there was a more frequent wish for reconstructive treatment in the group below 80% HPF symmetry. Adjusting the vertical palpebral fissure height to the horizontal palpebral fissure length only lowered the naturalness and increased the desire for intervention. This implies that further opening of the eye is in favor of the cosmetic appearance.

For the amount of enophthalmos (6 mm), one study was found where the degree of enophthalmos was studied on 12 “straight” photographs by 50 observers working at the ophthalmology department.^30^ This study found an enophthalmos of 5 mm to be disturbing, between 3 and 4 mm enophthalmos became “detectable”; and 2 mm or less was found to be “normal.” Another study evaluated 60 clinical photographs of patients with posttraumatic enophthalmos with 1 mm increments from 0 to 5 mm, using laymen as observers. This study found that enophthalmos smaller than 5 mm was unidentified by the observers.^31^ This corresponds with our study, where we, however, did not include the step of 5 mm displacement. This is also the only study that evaluated cosmesis from different angles using the videos, whereas other studies only used still photographs from different angles.

No other studies were found for the threshold for the caudal displacement of the eye and brow (2 mm). This study thus provides insight for the first time into what the position of the brows and eyes mean for the perception of naturalness.

Caudal positioning of the eye worsens the naturalness score; between 1 and 2 mm the score altered the most. Caudal positioning of the eye and brow together indifferently changed the perception of naturalness. We did not score the downward displacement of the brow alone, as we did not see disturbing examples of only brow involvement without (artificial) eye displacement in our cohort.

A strength of this study is that we have used a 3D animation with the use of raytracing. This allowed us to evaluate and assess the asymmetries under standardized conditions but with different shadow positions and cornea reflexes. Another strength is the originality of the study design and the direct clinical implications it has for daily practice in the care for MICA children. A possible flaw could be that we only used 1 face, and the implications for this particular face are not necessarily the same for every person. Additionally, MICA children sometimes express clinical features that were not modeled (such as shorter brows, larger intercanthal distance, entropion, and hemifacial microsomia with upward angulated nostrils and mouth), but we chose the main features that were most striking to us and that resulted in a realistic amount of videos to judge.

Further research needs to be done to evaluate in what ways the asymmetries influence each other and if thresholds are variable with different facial expressions.

## CONCLUSION

Enophthalmos of 6 mm or more, smaller horizontal palpebral fissures of ≤79%, and more than 2 mm downward brow and eye displacement resulted in a significantly different judgment from the normal version and a large increase in the desire for correction. This would mean that smaller asymmetries are not perceived as disturbing compared to a perfectly symmetrical face. Correcting asymmetries may therefore be less beneficial if these thresholds are not exceeded. These values can be used in decision-making or as an outcome measure in the treatment of congenital anophthalmia and microphthalmia.
